# Current diagnosis and treatment of chronic subdural haematomas

**Published:** 2015

**Authors:** IA Iliescu

**Affiliations:** * Department of Neurosurgery, University Hospital Bucharest, Romania

**Keywords:** chronic subdural haematoma, diagnostic, treatment

## Abstract

A developed society is usually also characterized by an elderly population, which has a continuous percentage growth. This population frequently presents a cumulus of medical pathologies. With the development of the medication and surgical treatment of different affections, the life span has increased and the pathology of an old patient has diversified as far as the cumulus of various pathological diseases in the same person is concerned. Chronic subdural pathologies represent an affection frequently met in neurosurgery practice. Any neurosurgeon, neurologist and not only, has to be aware of the possibility of the existence of a chronic subdural haematoma, especially when the patient is old and is subjected to an anticoagulant or antiaggregant treatment, these 2 causes being by far the etiological factors most frequently met in chronic subdural haematomas. With an adequate diagnosis and treatment, usually surgical, the prognosis is favorable. Although the surgical treatment presents a categorical indication in most of the cases, the fact that there are many surgical techniques, a great relapse rate, as well as the numerous studies, which try to highlight the efficiency of a technique as compared to another, demonstrate that the treatment of these haematomas is far from reaching a consensus among the neurosurgeons. The latest conservatory treatment directions are still being studied and need many years to be confirmed.

**Abbreviations**:

CT = computerized tomography, MRI = magnetic resonance imaging

## Definition

Subdural haematoma represents an extracerebral blood collection, which can be met as a clot or in liquid form, located between the dura mater and the middle layer of the meninges (arachnoid) and, which does not expand in the subarachnoid area or in the basal cisterns (interpeduncular cisterns). Usually, this collection has a traumatic etiology (the acute and subacute ones are always traumatic) and a compressive effect on the brain, producing localization neurological signs, increased intracranial pressure signs and various alterations of the consciousness. 

According to the time passed from the trauma to the one of the manifestation, they can be classified into the following groups: 

• Acute subdural hematoma – the manifestations appear during the first 3 days

• Subacute subdural hematoma – clinically manifests between 4 and 21 days 

• Chronic subdural hematoma – the clinical manifestations appear after 21 days 

It was first described by Virchow, in 1857, as “an internal hemorrhagic pachymeningitis”. Later, in 1914, Trotter launched the theory of traumatic brain injury and the consecutive lesion of the “bridging veins”, as being the cause of what he called “hemorrhagic subdural cyst”. 

## Epidemiology

With an approximate incidence of 2-3% presence in the neurosurgery clinics, subdural haematomas usually have a traumatic etiology and are most often produced due to falls from the same or from another level. The trauma is usually minor, with an anteroposterior direction and it causes the rupture of the emissary veins to the superior saggital sinus. In traumatic brain injury, between the acceleration and deceleration of the skull and the encephala there is a short interval, the brain following in a short time the movement or the stopping of the skull. The moment the skull stops, the brain, which has inertia, continues its movement while determining the stretching and subsequently the rupture of the bridging veins in the subdural space. If the traumas have a lateral direction, the subdural haematoma can form rarely, in which case besides the subdural haematoma there are also brain contusions or brain dilacerations.

The incidence of chronic subdural hematomas is estimated at 1,7-18/ 100.000 inhabitants of the entire population and it raises to 58/ 100.000 inhabitants in the group of patients over 65 years old. The medium age of patients with chronic subdural haematoma is of 63 years old. Due to the fact that the population continues to get old, it is expected that in 2030, its incidence will double [**1**-**4**]. Moreover, the prevalence of chronic subdural haematoma is of 69% vs. 31% in the age group of over 65 years old compared to the rest of the age groups. The incidence on sex highlights the fact that only the male population is more frequently affected – 64% vs. 33% according to some studies, 71,8% vs. 28,2% according to other studies, etc.

As far as the localization of chronic subdural haematomas are concerned, the studies have demonstrated a higher frequency of chronic subdural haematomas of left brain hemisphere (52%) compared to the ones of right brain hemisphere (30%), in 18% of the cases being bilateral.

What is interesting to mention is the medium thickness of chronic subdural haematomas, which was of approximately 20,5mm +/- 5mm in unilateral haematomas and of approximately 29,6mm +/- 9mm in bilateral haematomas. 

In 77% of the cases, the patients have suffered a trauma by falling and 41% of them were under oral anticoagulant treatment or antiplatelets. 

The relapses of chronic subdural haematomas are reported between 2,3% and 33%, in 25% of the cases, haematomas being bilateral.

Considered for a long time a risk factor and not an etiological factor, the treatment with anticoagulants and antiplatelets has become an important subject to be discussed in the last couple of years, having a major complication in the appearance of subdural haematomas, especially the chronic ones. In addition, in the last 10 years, all the studies and statistics have shown it as a main etiologic factor in chronic spontaneous subdural haematomas or better said in non-traumatic haematomas and in their relapse, the anticoagulant and antiplatelet treatment. The statistics show that in non-traumatic subdural haematomas in 71% of the cases, there was an anticoagulant or antiplatelets treatment as compared to only 18% in the traumatic group, and the relapse was of 17% in this group of patients. 

## Clinical diagnosis

The diagnosis of a chronic subdural haematoma can present many difficulties taking into account the fact that it frequently appears in elders, who anyway have a higher or a lower degree of psychical disorders due to brain involution. Moreover, in the context of a usual brain injury, which can pass unobserved and may frequently be ignored, the chronic subdural haematoma has a latent period until the appearance of the clinical symptoms, the diagnosis presenting many errors. 

Therefore, the main element that can lead to the diagnosis of chronic subdural haematoma is the minor brain injury; however, it should not be forgotten that it is seriously taken into account only by some of the patients or their families. Besides the trauma, there are 3 elements that can contribute to the clinical diagnosis of a chronic subdural haematoma [**5**]: the signs of hemispheric brain damage or the foci (mainly the motor deficits and the speech disorders), psychical disorders which are most often met (chronic subdural haematoma being one of the causes of evolutive dementia in elders) and the symptomatic fluctuation. Besides these 3 elements, other relevant clinical signs have also been described in cases of haematoma: headache, drowsiness, signs of intracranial hypertension [**6**]. 

Generally, the clinical stage of chronic subdural haematoma orients towards an injury at the level of the brain hemisphere but does not help in the differential diagnosis of other brain injuries. 

Usually, a progressive slow expansive intracranial process or an ischemic stroke is suspected. The suspicion of expansive intracranial injury makes us perform a paraclinical investigation (CT or MRI), which makes the diagnosis clearer by evidencing the iso- or hypodense subdural collection. 

If the CT examination is currently very easily accessible, the diagnosis of chronic subdural haematoma has become quite simple, the main condition being that you only have to think about such a diagnosis. This is valid mostly in cases of elders, who progressively develop psychic disorders, representing the group of patients with chronic subdural haematoma. 

Regarding the differential diagnosis, it should not be forgotten that the clinical signs of a haematoma mimic an intracranial neoformation or an ischemic stroke. In youngsters, chronic subdural haematomas often manifest with epilepsy crises and behavior disorders, which are due to alcohol consumption. 

## Paraclinical diagnosis

The diagnosis of chronic subdural haematoma has evolved in the last 20-30 years together with the introduction of the CT scan, the MRI and the access of the patients to this type of explorations.

**The CT scan**

**Fig. 1:**
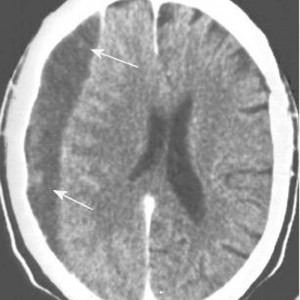
Chronic subdural haematoma of right brain hemisphere (CT scan); what should be noted is the hypodense aspect of the haematoma and the mass effect it has on the structures of the median line

**Fig. 2:**
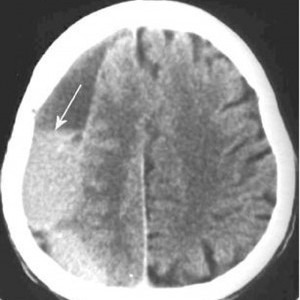
Chronic subdural haematoma with a recent bleeding of right brain hemisphere (CT scan); what should be noted is the mixed, hypo- hyperdense aspect of the haematoma and the erase of the circle movements of the right brain hemisphere as an effect of the compression due to the haematoma

It was introduced in 1972, based on the researches of Hounsfield. By using the densitometry differences at the level of the serial sections of the skull and its contents, it created the possibility of evidencing different injuries, the injured structure and the movement of the brain structures – the mass effect. It represents an investigation, which is currently very often used, it is rapid and it can also be applied in comatose patients, which has led to the precocious screening of many brain injuries. Being an extremely accessible investigation at present, which offers important information for the neurosurgeon in many cases of brain injuries, the CT scan is an essential tool in emergency brain injuries traumas. Currently, no emergency room of an emergency hospital can be conceived without having a CT scan (**Fig. 1**,**2**).

It was introduced in 1982 and it is based on the movement of the hydrogen atoms, which are included in different tissues in a magnetic field. In addition, each structure, even in the same organ, presents the specific T1 and T2 relaxation times, determining particular aspects of the whole MRI image. The intensity of the signal from different structures is captured and translated to an image with white shades, which means strong signal, or black shades, which means lack of signal. 

**Fig. 3:**
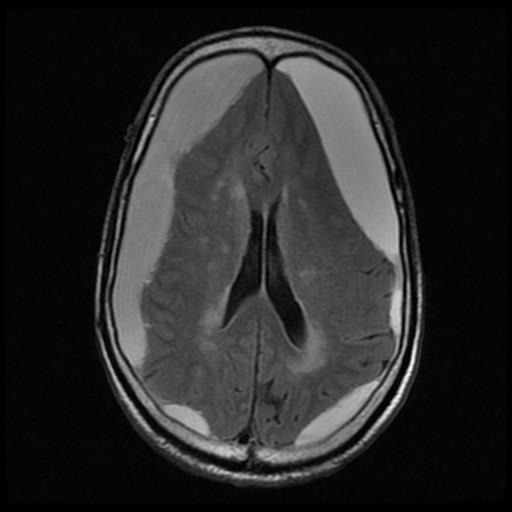
Bilateral chronic subdural haematoma; what should be taken into account is the absence of the mass effect on the structures of the median line, and the presence of the compressive effect on the noble brain structures – MRI image, T1

**Fig. 4:**
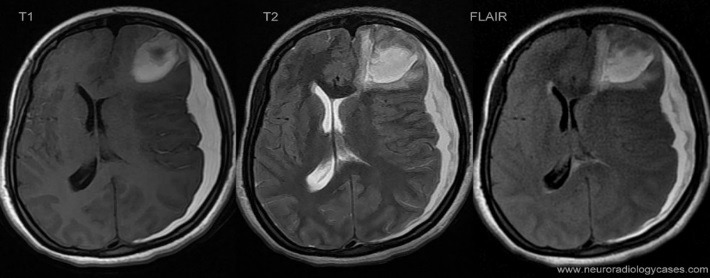
FLAIR, T1, T2 MRI images of an acute subdural haematoma of a left brain hemisphere which determines an important mass effect on the structures of the median line as well as the concomitant presence of an area of left frontal lobe laceration; complex brain injury

This way the cerebrospinal fluid, the bone, the rapid blood flow and the air, appear as being black or in different shades of black in T1. The cerebral gray matter and the white substance appear in T1 in different shades of gray. The fat and the recent blood effusions emit a strong signal, these being white in T1. In T2, the images are clearer, reflecting the modifications in the water content. In addition, the structures that contain a large amount of water appear with a strong signal, being white, as for example the cerebrospinal fluid (CSF), cerebral edema, demyelinated areas, tumors with a content characterized by a large amount of water. Due to the fact that the bone does not contain water, it appears as being black and its anatomical details are visualized harder than in a CT scan or in the images of an MRI in T1 (**Fig. 3**,**4**). 

Although it is an exceptional investigation tool, the MRI has its limitations which are given by the presence of some metallic structures in the organism as well as by the long process of images acquisition, which, in some cases, can make the examination impossible (for example the claustrophobic patients or the medico-surgical emergencies). 

As far as the traumatic brain injuries are concerned, the CT and the MRI have advantages and disadvantages when being compared. 

In addition, the CT examination is fast, more accessible than the MRI examination and brings essential information for the neurosurgeon in a short time. What should also be taken into account is that in a CT scan, a bone injury is easily and correctly appreciated. The MRI image presents a higher fidelity than the CT scan as far as the images and the injuries are concerned. It can also highlight the so-called diffuse axonal injuries of the brain (DAI); practically these could be described and studied closely only after the introduction of MRI examination. This type of injuries is predominantly located in the parasagittal cortical white-matter, the corpus callosum and the pontomesencephalic junction adjacent to the superior cerebellar peduncle. The microscopic aspect of this type of injuries corresponds to the Wallerian axonal degeneration so that the axon practically disintegrates.

There are studies concerning the chronic subdural haematomas, which also demonstrate the superiority of the MRI examination in comparison with the CT [**7**]. The MRI examination better shows the location of the chronic subdural haematoma and evidences its dimensions much clearer together with the mass effect of the adjacent structures. Moreover, it is more useful in cases of bilateral and isodense chronic subdural haematomas. The MRI examination is superior to the CT examination as far as the membranes dimensions of the chronic subdural haematoma and the presence of the septa inside the haematoma are concerned, in these conditions the surgical approach could be modified. 

## Treatment

Over the years, together with the development of the diagnosis methods and the surgical techniques, the treatment of chronic subdural haematomas has evolved; currently there are also studies of conservative treatment beside the various surgical techniques, which however have not been applied very often. 

In addition, as far as the surgical treatment is concerned, some techniques have been described: 

• minimal craniotomy (under 3cm diameter) technique – “single burr hole” – with a closed vacuum drainage system

• minimal craniotomy (under 3cm diameter) technique – with a closed drainage system and irrigation with sodium chloride solution (physiologic serum), which is a variant of the first technique 

• “twist drill trephination” technique – together with a closed drainage system 

• 2 minimal craniotomies technique – with a drainage system (a variant of the previous technique) 

• craniotomy (the diameter of the craniotomy being of over 3cm), together with a vacuum closed drainage system – usually used in cases of old, septated chronic subdural haematomas 

The 3 main surgical techniques used the treatment of chronic subdural haematomas consisted of the following:

1. Craniotomy – most often used in the past, it has the advantage of exposing an important part of the brain. It is usually practiced under general anesthesia and it is the most invasive of the treatment options of chronic subdural haematomas because it takes a lot of time and it presupposes a lot of intraoperatory blood loss. However, it remains the chosen option in cases of old, organized, multiseptated subdural haematomas. In Markwalder’s study (1981) on chronic subdural haematomas, craniotomy, which was considered a radical approach, was reserved to cases in which: a) the subdural haematoma relapsed; b) the haematoma seemed to be very well organized in the CT examination and having solid consistency; c) the brain did not re-expansion and obliterated the subdural area [**8**]. 

2. Minimal craniotomy (trephination) - is one of the most often used surgical techniques in cases of current chronic subdural haematomas. According to a national Canadian study in 2005, 85% of the respondents have indicated this technique as the most commonly used as initial surgical treatment. However, in cases of relapses, 43,1% preferred the craniotomy and only 35,1% the trephination [**9**]. It is usually done under general anesthesia, some surgeons preferring only one trephination, others two. Nevertheless, none of the 2 variants represents a clear choice although in a study it was shown that patients whom were applied only one trephination presented a high relapse rate, a longer hospitalization period and a high rate of infection of the wound [**10**].

3. Single twist drill trephination – presents the great advantage that it can be performed in the hospital bed while using local anesthesia or in the surgery room under local or general anesthesia. It is considered useful in cases of patients with multiple comorbidities, who are very old and in whom the risks of a complex surgery are very high. It is efficient only in cases in which the subdural haematoma is completely liquefied and, usually a Jackson-Pratt vacuum drainage system is also used. What should also be mentioned is that most of the studies highlight that the relapse rate in the case of a surgical treatment is between 5 and 33%. The relapse in cases of chronic subdural haematomas means the symptomatic relapse of the subdural haematoma in the area which was already operated on. The risk factors of relapses are the following: tendency of bleeding, intracranial hypotension, recurrent neomembrane hemorrhages. Moreover, the persistence of the subdural area due to the insufficient expansion of the brain is considered a risk factor for the recurrence of a haematoma. In case it is not symptomatic, it is not regarded as a recurrence. As far as the brain re-expansion is concerned, it is considered that its mechanical properties, such as the elasticity, can explain the tendency of its re-expansion. The factors of brain elasticity have been considered the compression the cerebrovascular volume, meningeal membranes and the subpial transection test [**11**]. Some studies highlight that the existence of neomembranes and the volume of primary brain expansion that precedes decompression, is not considered a predictor for the clinical aspect of the patient postoperatory.

According to a very thorough and on a long period of time study done in India, on a period of 30 years, which consisted of 2300 cases, the best technique was considered temporal craniotomy with the dura mater left open and the subdural area communicating with the subtemporal area. According to this study, the membranectomy was not necessary, the relapse rate diminished and the mortality rate was of only 0,5% [**12**].

In spite of these published results, the treatment of chronic subdural haematomas remains a controversy! Many neurosurgeons recommend a minimal craniotomy followed by an irrigation of the subdural area with sodium chloride solution and the use of a vacuum drainage system. Some studies even raise controversies regarding the utility of a drainage system.

Other neurosurgeons recommend the endoscopic treatment of chronic subdural haematomas, especially the septated ones [**13**,**14**].

It was also proposed that the universal technique should be the single twist drill trephination, followed by the use of a subdural catheter, instead of a minimal craniotomy, on a group of 66 patients. In this study, the reintervention rate was of 18,1% in the group of patients with a single twist drill trephination and of 33,3% in the one with minimal craniotomy [**15**]. 

A study that was recently published showed that in the absence of a clear evidence of the superiority of a certain type of surgical intervention, it is the neurosurgeon’s job to choose the technique to use: craniotomy or minimal craniotomy followed by drainage. The study was centered on the costs of each procedure, the relapse rate and reintervention, mortality and postoperatory morbidity. The conclusions of the study done on 119 patients, of whom 61 underwent minimal craniotomy and 58 craniotomy, are surprising: the percent of reintervention was lower in minimal craniotomy in comparison with craniotomy which had a 6,6% as compared with 24,1%, and the percent of postoperatory complications (infections, acute hemorrhages and stroke), was higher in patients with craniotomies. Regarding the medium hospitalization period, the medium period spent in the surgery room and the medium cost of the treatment, the minimal craniotomy followed by drainage also proved superior to craniotomy. Another conclusion of the authors, who have taken into consideration the costs of each type of intervention (craniotomy and minimal craniotomy), was that due to the fact that the health state of the population has a raising cost, the surgeons who choose to perform surgeries which are more expensive when there are others which are cheaper and more efficient, should be more careful when choosing the type of intervention to perform [**16**].

Another special category in the treatment of chronic subdural haematomas is the patients with anticoagulant treatment. This category is continually growing because a high proportion of the elder population undergoes a daily treatment with oral anticoagulants. A recent study has shown that the anticoagulant treatment raises to 42,5 times the risk of developing a chronic subdural haematoma [**17**], and another study done in Switzerland showed that 41% of the patients with chronic subdural haematomas undergo a daily anticoagulant treatment.

Besides the raise of the risk of developing a chronic subdural haematoma, coagulopathy also complicates the neurosurgical treatment. A study done in the U.S.A. showed that the patients undergoing an anticoagulant treatment present a medium hospitalization period higher than the others – 11 vs. 7,5 days. There is a consensus as far as the patients with anticoagulant medication who present symptomatic chronic subdural haematomas are concerned: they have to be readjusted from the point of view of coagulopathy in order to prevent the development of a haematoma and also to facilitate the neurosurgery. The initial treatment of coagulopathy is necessary even in patients with severe cardiac pathology with a specific indication of anticoagulants, as for example the patients with valvular prostheses.

The ones undergoing antiplatelets therapy represent another category of patients at risk. Also in their case, stopping the antiplatelets medication is the first step before any decision of performing a neurosurgery. The reintroduction of antiplatelets therapy can be done similar as in the case of anticoagulant therapy.

As far as the prophylactic anticonvulsive treatment is concerned, there are numerous pro and con opinions; the studies indicate rates between 2 and 19% of the patients who present epileptic crises pre- or post surgical intervention. The majority’s opinion is that the prophylactic administration of anticonvulsants is reasonable, mostly because the patients with chronic subdural haematomas frequently present other traumatic brain injuries, which are a well-known risk factor in crises appearance. However, what should be taken into account is the fact that the anticonvulsant medication represents an additional risk factor of falling from the same level in the population over 65 years old, also representing the main age group who presents chronic subdural haematomas. 

Two studies report there is no significant difference in the appearance rate of convulsive crises in patients who have received prophylactic antiepileptic treatment as compared to the ones who have not. However, both studies underline the fact that the benefit of administering prophylactic antiepileptic treatment is overtaken by the risks of their administration except for the patients with a high risk of presenting crises, such as the chronic alcohol consumers [**18**,**19**].

Besides the surgical techniques, conservative methods of treatment have been described and are still being studied: 

• tranexamic acid administration

• oral administration of corticosteroids 

The oral administration of tranexamic acid in a daily dose of 750mg has as basis the studies which indicate that fibrinolysis and the kinin-kallikrein inflammatory system have an important role in the growth of chronic subdural haematoma. These studies have led to the idea that the antifibrinolytic and anti-inflammatory treatment administration, basically tranexamic acid, can lead to the complete resection of the subdural haematoma. The study involving the tranexamic acid was conducted on a period of 4 years, the clinical follow up and the imagistic analysis of the patients in the study being on average of 58 days and the medium volume of the chronic subdural haematoma being reduced to 55,6ml. After the treatment with tranexamic acid, the volume of the haematoma was reduced to a medium size of 3,7ml, without its reoccurring or growing in size [**20**]. 

Another conservative treatment method is represented by the oral administration of corticosteroids (a double-blind multicentric randomized study, in Montpellier University Hospital, started in 2011), such as Prednisone in a dose of 1mg/ kg body/ day for 1 week, and then the dose is reduced with 10 mg/ week, until a dose of 5-10mg/ day is reached, a moment when the treatment can be stopped; the medium duration of the treatment being of 2 months. This treatment also has as a main idea the anti-inflammatory role medication administration with the purpose of counteracting the local inflammatory reaction at the level of the parietal membrane and consecutively the determination of the reduction in size of the haematoma. This study also proposed the administration of corticosteroids after surgery, with the purpose of preventing the relapses and a new reintervention, knowing the fact that the relapses in chronic subdural haematomas can reach to 30%. The study is still ongoing, no preliminary conclusions being drawn yet.

However, in a study done on 496 patients with chronic subdural haematoma, the administration of corticoids preoperatively has been associated with a decrease in the rate of relapses. The preoperatory use of corticoids did not determine a growth of complications or of mortality and morbidity rates in patients [**21**].

## Conclusion

Although the surgical treatment has a categorical indication in most of the cases, the fact that there are many surgical techniques, a higher relapse rate, as well as the multiple studies that try to highlight the efficiency of a technique over another, it demonstrates that the treatment of these haematomas is far from reaching a consensus among the neurosurgeons. However, it is obvious that the purpose of the treatment is to be minimally invasive and, in the same time, to diminish the rate of relapses. The new non-surgical treatment directions are still at the beginning and they need time to prove their efficiency.
